# Latent class analysis of obesity‐related characteristics and associations with body mass index among young children

**DOI:** 10.1002/osp4.414

**Published:** 2020-04-07

**Authors:** Laura N. Anderson, Ravinder Sandhu, Charles D.G. Keown‐Stoneman, Vanessa De Rubeis, Cornelia M. Borkhoff, Sarah Carsley, Jonathon L. Maguire, Catherine S. Birken

**Affiliations:** ^1^ Department of Health Research Methods, Evidence, and Impact McMaster University Hamilton Ontario Canada; ^2^ Division of Child Health Evaluative Sciences (CHES) Sick Kids Research Institute Toronto Ontario Canada; ^3^ Applied Health Research Centre of the Li Ka Shing Knowledge Institute of St. Michael's Hospital University of Toronto Toronto Ontario Canada; ^4^ Division of Biostatistics, Dalla Lana School of Public Health University of Toronto Toronto Ontario Canada; ^5^ Institute of Health Policy, Management, and Evaluation, Faculty of Medicine University of Toronto Toronto Ontario Canada; ^6^ Department of Health Promotion, Chronic Disease and Injury Prevention Public Health Ontario Toronto Ontario Canada; ^7^ Division of Pediatric Medicine and the Pediatric Outcomes Research Team (PORT), Department of Pediatrics Hospital for Sick Children Toronto Ontario Canada; ^8^ Department of Nutritional Sciences, Faculty of Medicine University of Toronto Toronto Ontario Canada; ^9^ Department of Pediatrics St. Michael's Hospital Toronto Ontario Canada

**Keywords:** child obesity, latent class analysis, nutrition, physical activity

## Abstract

**Objective:**

Identifying how obesity‐related characteristics cluster in populations is important to understand disease risk. Objectives of this study were to identify classes of children based on obesity‐related variables and to evaluate the associations between the identified classes and overweight and obesity.

**Methods:**

A cross‐sectional study was conducted among children 3–11 years of age (*n* = 5185) from the *TARGet Kids!* network (2008–2018). Latent class analysis was used to identify distinct classes of children based on 15 family, metabolic, health behaviours and school‐related variables. Associations between the identified latent classes and overweight and obesity were estimated using multinomial logistic regression.

**Results:**

Six classes were identified: Class 1: ‘Family and health risk behaviours’ (20%), Class 2: ‘Metabolic risk’ (7%), Class 3: ‘High risk’ (6%), Class 4: ‘High triglycerides’ (21%), Class 5: ‘Health risk behaviours and developmental concern’ (22%), and Class 6: ‘Healthy’ (24%). Children in Classes 1–5 had increased odds of both overweight and obesity compared with ‘Healthy’ class. Class 3 'High risk' was most strongly associated with child overweight (odds ratio [OR] 1.9, 95% confidence interval [CI] 1.2, 3.2) and obesity (OR 3.3, 95% CI 1.7, 6.7).

**Conclusions:**

Distinct classes of children identified based on obesity‐related characteristics were all associated with increased obesity; however, the magnitude of risk varied depending on number of at‐risk characteristics. Understanding the clustering of obesity characteristics in children may inform precision public health and population prevention interventions.

## 1 INTRODUCTION

Overweight and obesity in childhood are complex conditions with multiple risk factors and sequelae. Body mass index (BMI)‐defined cut‐points are the most commonly reported measure of overweight and obesity; however, BMI alone may not adequately capture the complex nature of obesity as a disease and may not lead to effective interventions for prevention or treatment.^1^, ^2^ Clinical staging tools such as the Edmonton Obesity Staging System for paediatrics classify the severity and functional limitations related to obesity according four domains (Metabolic, Mechanical, Mental Health and Social Milieu)^3^, ^4^ but are not well suited to large population surveys because many the factors require clinical assessment. Other common classifications of obesity are based on the presence or absence of metabolic risk factors (e.g. metabolically healthy obesity).^5^ In contrast, there are normal weight children with obesity‐related risk factors and complications in the population who are not considered to be overweight or have obesity based on BMI.^6^ Understanding the clustering of obesity‐related variables in young children and investigating how such clusters are related to BMI‐defined overweight and obesity may have important implications for improving obesity prevention and management through targeted interventions.

Obesity‐related variables defined broadly to include both risk factors (e.g., poor diet, low physical activity and family history of obesity) and complications or consequences of obesity (e.g., increased cholesterol, blood pressure and behavioural problems), are generally considered in isolation. A 2014 review paper identified 18 studies on the clustering of diet and physical activity in children, and only three studies used latent class analysis (LCA).^7^ LCA is a data‐driven analytical method that groups heterogeneous populations into homogeneous groups based on categorical indicator variables and has some advantages over other cluster analysis methods.^8^, ^9^ In addition to the three studies^10^, ^11^, ^12^ identified in the previous review, three other studies^13^, ^14^, ^15^ that used LCA in children were identified—all of these studies were conducted in children >9 years of age and focused primarily on diet and physical activity. These six previous LCA studies^10^, ^11^, ^12^, ^13^, ^14^, ^15^ each identified between three to six specific classes (e.g., healthy, sedentary, physically active and health risk behaviour), and each of these classes were differentially associated with overweight and obesity.

Thus, the overall aim of this study was to identify distinct classes of children, as young as 3 years of age, based on a comprehensive range of obesity‐related variables, including family, metabolic, health behaviours and developmental concerns. The primary objective of this study was to identify distinct groups of healthy children 3 to 11 years of age based on obesity‐related variables, overall and stratified by sex. The secondary objective was to evaluate the association between the identified latent classes and BMI categories (normal weight, overweight and obesity).

## 2 METHODS

### 2.1 Study design

A cross‐sectional study was conducted using data from The Applied Research Group for Kids (TARGet Kids). TARGet Kids is a primary care research network in Toronto, Canada. Children <6 years of age were recruited between 2008 and 2018 during well‐child visits at primary care paediatric and family physician practices in the Greater Toronto Area and are followed annually.^16^ Exclusion criteria at enrolment were <32 weeks gestational age, growth‐restricting health conditions such as cystic fibrosis or failure to thrive, severe developmental delay, or other chronic conditions (not including asthma and high functioning autism) and non‐English speaking parents.^16^ For this cross‐sectional study, children with a visit between 3 to 11 years of age (36 to <144 months) were included. Children <3 years of age were excluded because blood pressure was not measured in this age group.^16^ Children ≥12 years of age were excluded because there were relatively few children of this age in TARGet Kids, and this study was specifically interested in younger children as obesity interventions may be more successful at younger ages.^17^ For children with multiple visits in the 3‐ to 11‐year age range, the first visit with a blood sample was selected; for children who never provided a blood sample, their first visit with questionnaire data within this age range was used.

Trained research assistants recruited study participants and physical measurements, including anthropometrics and blood pressure, blood samples (nonfasted) and parent‐completed nutrition and health questionnaires were collected at each visit.^16^ Blood samples were processed by Mount Sinai Services Laboratory at Mount Sinai Hospital, Toronto, Canada, and all data are stored and managed at the Applied Health Research Centre of the Li Ka Shing Knowledge Institute, St. Michael's Hospital, Toronto, Ontario. Written consent was obtained from parents, and Research Ethics Board approval was obtained from both The Hospital for Sick Children and St. Michael's Hospital, Toronto, Ontario.^16^ The *TARGet Kids!* cohort study is registered at www.clinicaltrials.gov (NCT01869530).

### 2.2 Measurement of body mass index *z*‐scores

Height and weight anthropometric data were collected by trained research assistants using a precision digital scale (seca, Germany) for weight and a stadiometer (seca) for measuring standard height. BMI was calculated by dividing weight (in kilogrammes) and height (in metres squared)^16^ and BMI *z*‐scores (zBMI) were calculated using the World Health Organization (WHO) growth standards for children under five and growth reference for children over five as recommended for the Canadian population.^18^, ^19^ For the purpose of this study and to conduct analysis combining all children from 3 to 11 years of age, the labels recommended for children over 5 years of age were consistently applied to all the children in this study: zBMI ≤ 1 was defined as ‘normal weight’; 1 < zBMI ≤ 2 was defined as ‘overweight’; zBMI > 2 was defined as ‘obesity’.^18^

### 2.3 Measurement of obesity‐related characteristics

Based on previous literature, including the domains identified in the Edmonton Obesity Staging System for Pediatrics,^3^ and in consideration of available data, 15 unique obesity‐related variables were selected a priori for inclusion as indicator variables in the LCA. It was not possible to directly use all of the same variables from the Edmonton Obesity Staging System because many of the clinical variables were not available in our study. These variables were broadly grouped into four categories for descriptive purposes only, however, these descriptive groupings did not inform the analysis (all variables were treated independently in the LCA). The four categories of variables were: (1) Family characteristics (family history of cardiometabolic disorders, parental overweight or obesity and low or mid parental income); (2) Cardiometabolic characteristics (low high‐density lipoprotein [HDL], high low‐density lipoprotein [LDL], high non‐HDL, high triglycerides, high blood pressure); (3) Health risk behaviours (low physical activity, high screen time, low time spent sleeping and high intake of sugar sweetened beverages [SSBs]); and (4) Developmental concerns (developmental or learning issues, requiring extra resources provided by school and concerns expressed by child's school). All 15 indicator variables were categorized into binary latent class indicators; coded as ‘1’ if they exhibited the at‐risk characteristic and ‘0’ if not at‐risk.

*Family characteristics* included family history of cardiometabolic disorder defined as ‘yes’ if a mother, father or siblings reported diagnosis of heart disease, hypertension, high cholesterol or diabetes versus ‘no’ if none reported. Parental BMI was calculated based on measured height and weight of either the mother or father collected by the research assistant at the child's primary care visit.^16^ For this study, parental BMI was 83% from mothers and 17% from fathers. Parental BMI ≥ 25 was defined as overweight or obesity versus BMI < 25.^20^ Low income was categorized as median neighbourhood income <$50,000 per year versus ≥$50,000 per year. Low income was defined as <$50,000 based on the low‐income measure threshold for before‐tax income for a family of four in Toronto, which was $51 031 in 2015.^21^

*Cardiometabolic characteristics* for children included high systolic blood pressure (SBP) or diastolic blood pressure (DBP), measured by trained research assistants according to recommendations,^22^ and dichotomized as high versus low based on the age‐specific guidelines (SBP or DBP ≥ 90th percentile for sex, age and height).^23^ Abnormal cholesterol was defined from nonfasting blood samples as described previously.^24^ The following cut‐offs were used to define abnormal lipid levels: HDL ≤ 1.17 mmol/L, LDL ≥ 2.85 mmol/L), non‐HDL ≥ 3.11 mmol/L and triglycerides (0–9 years ≥0.85 mmol/L, TG (10–19 years) ≥ 1.01 mmol/L).^25^

*Health risk behaviours* included parent‐reported physical activity, screen time, sleep and sugar‐containing beverages (SCBs). The measurement of each of these variables is described in more detail elsewhere.^16^ Physical activity was measured as weekday freeplay, and children were classified as not meeting physical activity recommendations if they had less than 180 min/day of unstructured free play (under 5 years), or less than 60 min per day (5 years and over).^26^, ^27^ Sedentary behaviour was estimated by screen time in reference to how much time the child spent awake using a TV, DVD, video game or mobile device on a typical weekday. Children under five that reported greater than 60 min per day of screen time, and children 5 years and older that reported greater than 120 min per day were identified as having high screen time.^27^, ^28^ Parent‐report of child sleep duration was dichotomized as not meeting recommendations if sleep duration was <10 h for children 3–5 years or <9 h for children 6–13 years, versus meeting or exceeding recommendations.^29^ SCBs were calculated as the number of cups of 100% juice, sweetened drinks and soda or pop the child consumed on a typical day. Based on recommendations outlined by the American Academy of Pediatrics, children were categorized as SCB intake not meeting recommendations if they consumed >0.5 cup/day for age less than 3, >0.75 cup/day for age 4–6, and >1 cup/day for ages greater than 7.^30^

*Developmental concerns included* children that were reported to have been diagnosed with attention deficit hyperactivity disorder, autism, learning problem or developmental delay on the Nutrition and Health Questionnaire (NHQ) that were identified as having developmental concern. Children were identified as having school concerns if parents reported that their child's school expressed concerns about the child relating to the following: speech and language, learning, attention, behaviour, social relationships, physical coordination, fine motor coordination or self‐help skills and independence. Children were identified as requiring extra resources if parents reported the provision of one or more of the following extra resources from their child's school: speech and language therapy, occupational therapy, educational assistance or other extra resources provided at school.

### 2.4 Statistical analysis

Descriptive analyses of covariates including sex, age and weight category were examined for participants, as well as the frequency of responses for each of the 15 latent class indicators across all participants in the sample. For the primary objective, LCA was conducted to identify unobserved (latent) classes based on the categorical indicators described.^31^ LCA assigns each participant a ‘best’ class assignment based on their probability of belonging to each class. Participants within the same class are considered to be homogeneous based on the indicator variables.^8^

Analyses were conducted using the LCA procedure in SAS version 9.4 software.^9^ Based on previous studies, models with 1–8 classes were tested to select the optimal number of classes. No covariates were included in this procedure. To determine the optimal number of classes, consistent Bayesian information criterion (BIC) and Akaike information criterion (cAIC) values for each model were compared, and the model with the lowest values was the best fit. The distributions of the item response probabilities were evaluated, and the identified classes were named based on which characteristics were more likely to be exhibited by members of the class. Participants were assigned to the class in which they had the highest probability of membership; that is, they exhibited the traits that are representative of that class. The associations between the identified latent classes and zBMI categories were then evaluated using multinomial logistic regression adjusted for child age and sex. Age and sex‐adjusted odds ratios (ORs) and 95% confidence intervals (CIs) were reported. All analyses were conducted overall (among males and females combined) and sex stratified.

Under the assumption that missing data was missing at random (MAR), the model was fit using an Expectation–Maximization (EM) approach, where all available data was used to estimate classes and assign the posterior probabilities of belonging to each class for each individual.^9^ All individuals were assigned to a class using this approach, and this class was then used in the regression analysis resulting in no missing data for the final regression analysis.

## 3 RESULTS

### 3.1 Participant characteristics

A total of 5185 children between 3 and 11 years of age were included in this study; 74% of children were in the 3‐ to <6‐year age range. As described in Table 1, 2471 (48%) children were female, and 2714 (52%) were male. The mean age was 61 months (5 years) with a standard deviation of 21.1 months. Based on zBMI, 81% of children were considered ‘normal’ weight; 14% had overweight, and 5% had obesity; the proportion of children with obesity was slighter higher in males than females. The frequency of all obesity‐related characteristics included as indicators in the LCA are also provided in Table 1. About half of the children (48%) did not provide a blood sample and thus had no lipid measures.

**TABLE 1:** Descriptive characteristics and distribution of latent class indicators (*N* = 5185)

Variables	All children (*n* = 5185)	Male (*n* = 2714)	Female (*n* = 2471)
Participant characteristics			
Age (years), mean (SD)	5.0 (1.8)	5.1 (1.8)	5.0 (1.8)
3 to <6 years	3810 (74%)	1991 (74%)	1819 (74%)
6 to <9 years	1157 (22%)	598 (22%)	559 (23%)
9 to <12 years	218 (4%)	125 (5%)	93 (4%)
Sex			
Male	2714 (52%)	2714 (100%)	
Female	2471 (48%)		2471 (100%)
Maternal ethnicity			
European	3238 (67%)	1695 (68%)	1541 (67%)
East, South or Southeast Asian	865 (18%)	457 (18%)	408 (18%)
Other	714 (15%)	354 (14%)	357 (15%)
Maternal education			
College or university	4604 (91%)	2400 (90%)	2200 (92%)
High school or less	459 (9%)	253 (10%)	204 (8%)
BMI *z*‐scores			
zBMI ≤1 (normal weight)	4122 (81%)	2119 (79%)	2003 (83%)
1 > zBMI≤1 (overweight)	735 (14%)	400 (15%)	335 (14%)
zBMI>2 (obesity)	230 (5%)	103 (6%)	60 (3%)
Missing	98	47	51
Indicator variables	N (%)	N (%)	N (%)
Family history of cardiometabolic disorders			
No	3520 (81%)	1826 (80%)	1694 (81%)
Yes	851 (19%)	445 (20%)	406 (19%)
Missing	814	443	371
Parental BMI*			
Normal (<25 kg/m^2^)	2622 (56%)	1365 (56%)	1257 (57%)
Overweight and obese (≥25 kg/m^2^)	2020 (44%)	1080 (44%)	940 (43%)
Missing	543	269	274
Neighbourhood household income			
≥$50,000	3092 (64%)	1606 (64%)	1486 (65%)
<$50 000	1715 (36%)	898 (36%)	817 (35%)
Missing	378	210	168
Blood pressure			
Meeting recommendations	3291 (78%)	1732 (78%)	1559 (77%)
High SBP and/or DBP	931 (22%)	477 (22%)	454 (23%)
Missing	963	505	458
HDL			
Meeting recommendations	2117 (78%)	1148 (79%)	969 (77%)
<1.17 mmol/L	598 (22%)	310 (21%)	288 (23%)
Missing	2470	1256	1214
LDL			
Meeting recommendations	2408 (89%)	1318 (91%)	1090 (87%)
> 2.85 mmol/L	299 (11%)	136 (9%)	163 (13%)
Missing	2478	1260	1218
Non‐HDL			
Meeting recommendations	2129 (78%)	1176 (81%)	953 (76%)
> 3.11 mmol/L	584 (22%)	281 (19%)	303 (24%)
Missing	2472	1257	1215
Triglycerides			
Meeting recommendations	1085 (40%)	598 (41%)	487 (39%)
Not meeting recommendations (age‐based)	1632 (60%)	861 (59%)	771 (61%)
Missing	2468	1255	1213
Physical activity			
Meeting recommendations	894 (19%)	502 (20%)	392 (17%)
Not meeting guidelines (age‐based)	3871 (81%)	2000 (80%)	1871 (83%)
Missing	420	212	208
Screen time (sedentary)			
Meeting recommendations	2254 (55%)	1149 (53%)	1105 (57%)
Not meeting guidelines (age‐based)	1863 (45%)	1037 (47%)	826 (43%)
Missing	1068	528	540
Time spent sleeping			
Meeting recommendations	4068 (88%)	2156 (89%)	1912 (88%)
Below recommendations (age‐based)	532 (12%)	267 (11%)	265 (12%)
Missing	585	291	294
SSB intake			
Meeting recommendations	2635 (57%)	1347 (56%)	1288 (58%)
Not meeting guidelines (age‐based)	2014 (43%)	1076 (44%)	938 (42%)
Missing	536	291	245
Developmental/learning issue reported			
None	4420 (95%)	2250 (93%)	2170 (97%)
At least one reported	236 (5%)	168 (7%)	68 (3%)
Missing	529	296	233
Extra resource at school			
None	2334 (72%)	1183 (68%)	1151 (76%)
Requires at least one	929 (28%)	562 (32%)	367 (24%)
Missing	1922	969	953
Concern expressed by school			
None reported	2029 (62%)	969 (56%)	1060 (70%)
At least one concern	1234 (38%)	776 (44%)	458 (30%)
Missing	1922	969	953

Abbreviation: BMI, body mass index; DBP, diastolic blood pressure; HDL, high‐density lipoprotein; LDL, low‐density lipoprotein; SBP, systolic blood pressure; SSB, sugar sweetened beverage.

* Parent BMI measures were 83% from mothers and 17% from fathers.

### 3.2 Latent class analysis

**FIGURE 1:**
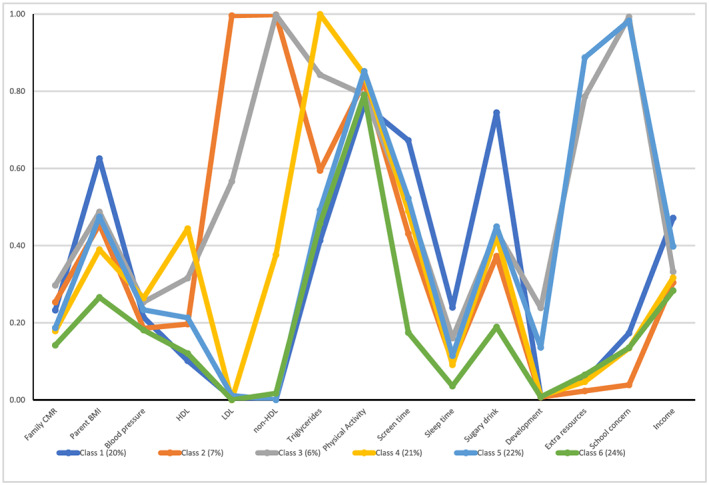
Graphical display of item response probabilities for each class (see Supporting Information Table S2 for numerical display)

For the primary analysis, a six‐class model was found to be the optimal model as it had lower cAIC and BIC values compared with the other models (Supporting Information Table S1). Response probabilities for each of the 15 indicators by class are presented graphically in Figure 1 (and numerically in Table S2). The 6 identified classes were named by the authors based on the indicators with a high response probability and included the following proportions of children: Class 1: ‘Family and health risk behaviours’ (20%), Class 2: ‘Metabolic risk’ (7%), Class 3: ‘High risk’ (6%), Class 4: ‘High triglycerides’ (21%), Class 5: ‘Health risk behaviours and developmental concern’ (22%), and Class 6: ‘Healthy’ (24%). Low physical activity was highly prevalent across all classes with approximately 80% of children in all classes (including the Healthy class) not meeting physical activity guidelines for their age group and thus is not discussed further as it did not help discriminate classes.

Class 6 was defined as the Healthy class as it had the lowest probability of almost all risk indicators compared with the other classes. Class 1 was defined as Family and health risk behaviours because children in this class were most likely to have parents with overweight or obesity, low neighbourhood household income, and not meet screen time or sugar beverage guidelines. Class 2‐labelled Metabolic risk was defined by a high probability of both high LDL and non‐HDL, in contrast, Class 4 was defined by high triglycerides only. Class 3 was considered a High risk class with high LDL, non‐HDL, triglycerides, screen time, extra school resources and school concern. In comparison, Class 5 was defined as Health risk behaviours and developmental concern only with high screen time, extra school resources and school concern.

### 3.3 Association of the latent classes with body mass index *z*‐score categories

**FIGURE 2:**
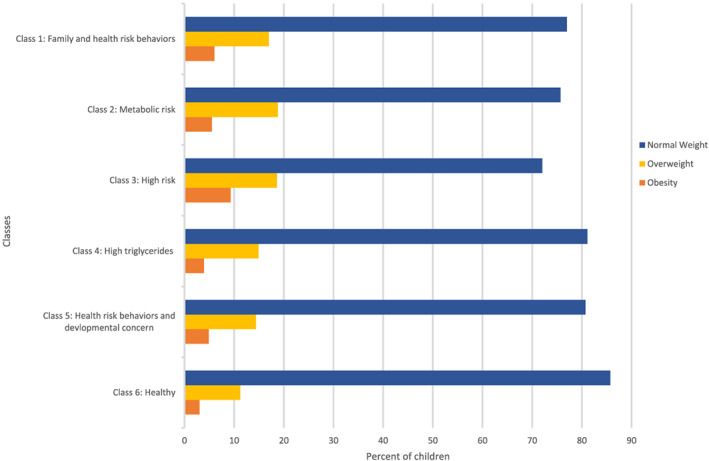
Distribution of body mass index *z*‐score (zBMI)‐defined weight categories by latent class

The distribution of zBMI categories across the six identified latent classes is presented in Figure 2. The proportion of children with overweight and obesity was highest in Class 3 High risk at 19% and 9%, respectively, and was lowest in Class 6 Healthy at 11% and 3%, respectively. Table 2 presents the results of the age and sex‐adjusted multinomial regression analysis for the association between each of the six identified latent classes and zBMI categories overweight and obesity each compared with the normal weight group. All of the classes from 1 to 5 were associated with increased odds of both overweight and obesity, and the ORs were consistently stronger for obesity than for overweight. For example, Class 3 High risk had the highest odds of increased overweight (OR 1.94, 95% CI 1.18, 3.18), obesity (OR 3.33, 95% CI 1.66, 6.68), compared with Class 6 Healthy.

**TABLE 2:** Adjusted odds ratio (OR) and 95% confidence intervals (CIs) of having overweight, and obesity by latent class for the total sample

Class	Overweight		Obesity	
	OR^*^	95% CI	OR^*^	95% CI
Class 1: Family and health risk behaviours	1.64	1.31–2.04	2.28	1.56–3.31
Class 2: Metabolic risk	1.86	1.27–2.71	2.24	1.16–4.32
Class 3: High risk	1.94	1.18–3.18	3.33	1.66–6.68
Class 4: High triglycerides	1.35	1.06–1.71	1.46	0.94–2.25
Class 5: Health risk behaviours and developmental concern	1.31	1.03–1.67	1.67	1.10–2.53
Class 6: Healthy	1.0		1.0	

* OR are adjusted for age and sex.

### 3.4 Sex‐stratified analysis

**FIGURE 3:**
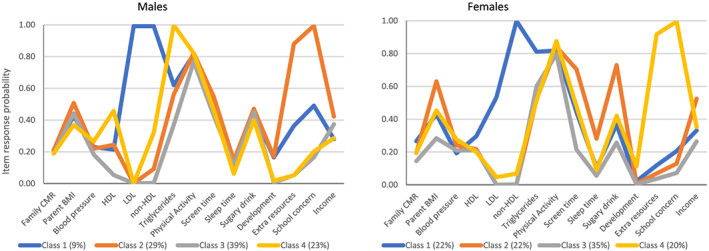
Graphical display of item response probabilities for each class for males and females separately (see Supporting Information Tables S4 and S6 for numerical display

Results of the sex‐stratified analyses revealed that a four‐class model fit the data best for the LCA in both males and females separately (model fit indices are provided in Tables S3 and S5). The item response probabilities for each of the 15 indicators by class for males and females are presented graphically in Figure 3 (and numerically in Tables S4 and S6). For both male and female children, the first three identified classes were somewhat similar between both males and females and were similar to the overall analysis with males and females combined; however, there were differences between males and females in the fourth class. Class 1 was defined Metabolic risk due to high LDL, non‐HDL and triglycerides, and included 9% of children among males but 22% of females. Class 2 was defined High risk and in males included 29% of children and was defined based on high triglycerides, screen time, extra school resources and concern at school, whereas in females this class included 22% and was defined as by high parent BMI, low neighbourhood income, high triglycerides, screen time and SSB (not school resources or concern). Class 3 was the Healthy class observed in 39% of males and 35% of females; in males, this class exhibited no high risk characteristics (except low physical activity which was observed in across every class as discussed previously), but in females, this class also had relatively high probability of high triglycerides. Class 4 in males (23%) was defined as High triglycerides only, whereas Class 4 in females (20%) was defined as ‘Developmental concern’ with a high probability of both extra resources required at school and school concerns.

Table 3 presents the results of the multinomial regression analysis for the association between each identified latent class and overweight and obesity stratified by sex. Among males, Classes 1 Metabolic risk and 2 High risk, compared with Class 3 Healthy, were associated with increased odds of overweight and obesity and Class 4 High triglycerides was associated with increased odds of overweight only. Similarly, in females, Classes 1 Metabolic risk and 2 High risk were associated with increased odds of overweight and obesity. Class 4 Developmental concern was not associated with overweight or obesity in females.

**TABLE 3:** Adjusted odds ratio (OR) and 95% confidence intervals (CIs) of having overweight, and obesity by latent class for males and females separately

Males	Overweight		Obesity	
	OR^*^	95% CI	OR^*^	95% CI
Class 1: Metabolic risk	1.95	1.28–2.98	1.90	1.00–3.62
Class 2: High risk	1.35	1.04–1.75	1.50	1.01–2.21
Class 3: Healthy	1.0		1.0	
Class 4: Triglycerides only	1.43	1.04–1.96	1.07	0.62–1.87
Females				
Class 1: Metabolic risk	1.47	1.06–2.02	2.21	1.21–4.09
Class 2: High risk	1.56	1.17–2.07	1.56	1.17–2.07
Class 3: Healthy	1.00		1.00	
Class 4: Developmental concern	0.89	0.38–1.08	0.89	0.61–1.32

* Adjusted for child age.

## 4 DISCUSSION

Results of this study suggest that there are distinct classes of young children defined based on presence of obesity‐related risk factors, and several of these classes were differentially associated with odds of overweight and obesity. A total of six classes were identified, and only one class (Class 6 Healthy), which included 24% of children, was defined by the absence of any obesity‐related risk factors (except for low physical activity that was highly prevalent across all classes). The other five latent classes, which included 76% of the children, all exhibited at least two obesity‐related characteristics. Of concern were the 6% of children identified in Class 3 with multiple obesity‐related characteristics, including family, metabolic, health risk behaviours and developmental concerns.

Each of the identified classes, compared with the healthy class as reference, was strongly associated with increased odds of overweight, and obesity in a consistent dose–response manner with much stronger associations for obesity than overweight. However, the magnitude of the strengths of the associations varied by class. Classes 1–3 defined as Family and health risk behaviours, Metabolic risk and High risk were significantly associated with substantial increases in odds of both overweight and obesity, with ORs ranging from 1.6 to 3.3.

Sex‐stratified analyses revealed that a LCA model with only four classes fit the data best for both boys and girls, compared with the six‐class model identified overall. Some sex‐differences were observed, but there were also many similarities between the four classes that were identified separately in males and females. Of the six previous LCA studies,^10^, ^11^, ^12^, ^13^, ^14^, ^15^ four studies evaluated sex‐differences.^11^, ^12^, ^13^, ^15^ Two of these studies^11^, ^12^ found that there were less girls in the high physical activity class compared with boys, while one study conversely found that girls were less likely to be in the high sedentary group compared with boys.^10^ This is consistent with a recent review that found that females were more likely to be in classes defined by low physical activity.^7^

Of the 15 indicator variables that were evaluated based on a priori hypothesis, the following five variables had little evidence of an association with any class membership: family history of cardiometabolic disorders, high systolic or diastolic blood pressure, abnormal HDL, sleep duration below age‐specific recommendations and parent‐reported developmental concern. In this study, low physical activity was also not a helpful indicator in determining the classes as it was highly prevalent in all classes (including the Healthy group); many children did not meet the recommended physical activity guidelines based on parent‐reported guidelines. This is contradictory to previous studies that found physical activity to be a determining characteristic for particular classes; however, this was in older children greater than 9 years of age.^10^, ^13^, ^14^, ^15^ Consistent with previous findings on clustering of risk behaviours,^13^ this study found that the same class could exhibit both risk and protective behaviours, such as low physical activity but also low sedentary behaviour (screen time) exhibited by all classes except Class 1 and 3.

Other studies that have used LCA to evaluate classes in older children, and adolescents did not incorporate metabolic risk factors such as blood pressure and lipid levels and instead focused only on dietary and behavioural risk factors.^10^, ^13^, ^14^, ^15^ Three of the identified classes, with a total of 34% of children, were defined by the presence of at least one metabolic risk factor (high LDL, non‐HDL or triglycerides). More work is needed to understand the rationale and implications of these distinct metabolic classes.

Strengths of the study include the relatively large sample size and comprehensive data available on family, metabolic, health risk behaviours and developmental concerns that were available for children participating in TARGet Kids!. This is one of the first studies to include such a wide variety of indicators in a cluster analysis, as well children as young as 3 years of age up to age 11. Additional strengths include the use of measured heights and weights and measurement of blood pressure by trained research assistants and blood collection for biomarker analysis. LCA also has several advantages compared with variable‐centred approaches, and it does not require a priori assumptions about the distribution of the data or the number of classes that will emerge.^32^

Children in this study were recruited from primary care providers in an urban setting and tended to have relatively high family income that may not be representative of all Canadian children. Missing data was substantial for some indicators because of changes in the study questionnaires over time, age‐specific measures and choice to provide blood samples. In the TARGet Kids cohort, it has previously been shown that characteristics of children who provided blood samples were similar to those who did not on most factors except age.^33^ LCA identifies class regardless of missing data, but this may have limitations if the data are not truly MAR. Many of the indicators are based on parent‐report and may be subject to measurement error and bias. For example, the validity of parent‐reported physical activity in this study is low to moderate when compared with objective measurement.^34^ There were other theoretically relevant indicators such as self‐esteem, school bullying and mental health that were not included because data were not available. Lastly, a potential limitation of LCA may arise from the dichotomization of continuous variables, and future studies may consider using an analytic approach that allows for the use of both dichotomous and continuous variables.

Population‐based interventions for the prevention^35^ and treatment^17^ of child obesity have demonstrated only modest success. The study results suggest that distinct classes of young children are defined based on obesity‐related risk factors, and the several classes are strongly associated with overweight and obesity. This may be important to consider when planning obesity prevention and treatment strategies. It is anticipated that these results may be most useful to population and public‐health providers and policy‐makers planning obesity interventions, but it is possible that this information would also be useful for clinicians when considering children who may benefit most from obesity prevention. Future studies are needed to determine how the clustering of obesity‐related characteristics may affect treatment. Further, future studies may consider including additional variables, particularly related to mental health and quality of life, to further understand the impact of these variables in defining the clustering of obesity‐related risk factors. Identifying classes of obesity‐related risk factors may help to inform public‐health efforts for obesity prevention and management.

## FUNDING INFORMATION

This study was supported by funding from the Canadian Institutes of Health Research.

## CONFLICT OF INTERESTS

JLM received an unrestricted research grant for a completed investigator‐initiated study from the Dairy Farmers of Canada (2011–2012), and Ddrops provided nonfinancial support (vitamin D supplements) for an investigator‐initiated study on vitamin D and respiratory tract infections (2011–2015). No other authors have any conflicts of interest to report.

## AUTHOR CONTRIBUTIONS

Laura N. Anderson, Cornelia M. Borkhoff, Sarah Carsley, Jonathon L. Maguire and Catherine S. Birken conceived the study and oversaw data collection. Laura N. Anderson, Ravinder Sandhu and Charles D.G. Keown‐Stoneman analysed the data. Laura N. Anderson and Ravinder Sandhu wrote the first draft, and all authors were involved in writing the final paper and approved the submitted version.

## Supporting information


**Table S1**: Model fit indices for 1 to 8 class LCA models
**Table S2**: Item response probabilities for the six class model overall (male and female children combined)
**Table S3**: Model fit indices for 1 to 8 class LCA models for males only
**Table S4**: Item response probabilities for the four‐class model for males only
**Table S5**: Model fit indices for 1 to 8 class LCA models for females only
**Table S6**: Item response probabilities for the four‐class model for females only

## Data Availability

Data are available upon request by contacting www.targetkids.ca/contact-us/. The full data are not freely available to respect the confidentiality of our participants, ensure data integrity and avoid scientific overlap between projects. Once initial contact has been made, we request a short research proposal that will be subject to review by the TARGet Kids! Scientific Committee and approval by institutional review boards.

## References

[1] Freedman DS , Wang J , Maynard LM , et al. Relation of BMI to fat and fat‐free mass among children and adolescents. Int J Obes (Lond). 2005;29:1‐8. 10.1038/sj.ijo.0802735 15278104

[2] Sharma AM , Kushner RF . A proposed clinical staging system for obesity. Int J Obes (Lond). 2009;33:289‐295. 10.1038/ijo.2009.2 19188927

[3] Hadjiyannakis S , Buchholz A , Chanoine J‐P , et al. The Edmonton Obesity Staging System for Pediatrics: a proposed clinical staging system for paediatric obesity. Paediatr Child Health. 2016;21:21‐26. 26941556 10.1093/pch/21.1.21 PMC4758422

[4] Hadjiyannakis S , Ibrahim Q , Li J , et al. Obesity class versus the Edmonton Obesity Staging System for Pediatrics to define health risk in childhood obesity: results from the CANPWR cross‐sectional study. Lancet Child Adolesc Health. 2019;3:398‐407. 10.1016/S2352-4642(19)30056-2 30952624

[5] Blüher S , Schwarz P . Metabolically healthy obesity from childhood to adulthood—does weight status alone matter? Metabolism. 2014;63:1084‐1092. 10.1016/j.metabol.2014.06.009 25038727

[6] Mokha JS , Srinivasan SR , DasMahapatra P , et al. Utility of waist‐to‐height ratio in assessing the status of central obesity and related cardiometabolic risk profile among normal weight and overweight/obese children: the Bogalusa Heart Study. BMC Pediatr. 2010;10:73 10.1186/1471-2431-10-73 20937123 PMC2964659

[7] Leech RM , McNaughton SA , Timperio A . The clustering of diet, physical activity and sedentary behavior in children and adolescents: a review. Int J Behav Nutr Phys Act. 2014;11:4 10.1186/1479-5868-11-4 24450617 PMC3904164

[8] Schreiber JB . Latent class analysis: an example for reporting results. Res Soc Adm Pharm RSAP. 2017;13:1196‐1201. 10.1016/j.sapharm.2016.11.011 27955976

[9] Lanza ST , Collins LM , Lemmon DR , Schafer JL . PROC LCA: a SAS procedure for latent class analysis. Struct Equ Model Multidiscip J. 2007;14:671‐694. 10.1080/10705510701575602 PMC2785099 19953201

[10] Huh J , Riggs NR , Spruijt‐Metz D , Chou C , Huang Z , Pentz M . Identifying patterns of eating and physical activity in children: a latent class analysis of Obesity Risk. Obesity. 2011;19:652‐658. 10.1038/oby.2010.228 20930718 PMC5310931

[11] Liu J , Kim J , Colabianchi N , Ortaglia A , Pate RR . Co‐varying patterns of physical activity and sedentary behaviors and their long‐term maintenance among adolescents. J Phys Act Health. 2010;7:465‐474. 10.1123/jpah.7.4.465 20683088

[12] Patnode CD , Lytle LA , Erickson DJ , Sirard JR , Barr‐Anderson DJ , Story M . Physical activity and sedentary activity patterns among children and adolescents: a latent class analysis approach. J Phys Act Health. 2011;8:457‐467. 21597117 10.1123/jpah.8.4.457 PMC3100677

[13] Fleary SA . Combined patterns of risk for problem and obesogenic behaviors in adolescents: a latent class analysis approach. J Sch Health. 2017;87:182‐193. 10.1111/josh.12481 28147457

[14] Laxer RE , Brownson RC , Dubin JA , Cooke M , Chaurasia A , Leatherdale ST . Clustering of risk‐related modifiable behaviours and their association with overweight and obesity among a large sample of youth in the COMPASS study. BMC Public Health. 2017;17:1‐11. 10.1186/s12889-017-4034-0 28109270 PMC5251243

[15] Iannotti RJ , Wang J . Patterns of physical activity, sedentary behavior, and diet in U.S. adolescents. J Adolesc Health off Publ Soc Adolesc Med. 2013;53:280‐286. 10.1016/j.jadohealth.2013.03.007 PMC3725190 23642973

[16] Carsley S , Borkhoff CM , Maguire JL , et al. Cohort profile: the applied research group for kids (TARGet Kids!). Int J Epidemiol. 2015;44:776‐788. 10.1093/ije/dyu123 24982016 PMC4521122

[17] Anderson LN , Ball GDC . Diet, physical activity, and behavioural interventions for the treatment of overweight or obesity in children and adolescents. Paediatr Child Health. 377‐382. 10.1093/pch/pxz006 PMC6735582 31528109

[18] WHO Child Growth Standards based on length/height, weight and age. Acta Paediatr Oslo Nor 1992 Suppl. 2006;450:76‐85. 10.1111/j.1651-2227.2006.tb02378.x 16817681

[19] de Onis M , Onyango AW , Borghi E , Siyam A , Nishida C , Siekmann J . Development of a WHO growth reference for school‐aged children and adolescents. Bull World Health Organ. 2007;85:660‐667. 10.2471/blt.07.043497 18026621 PMC2636412

[20] Health Canada . Canadian Guidelines for Body Weight Classification in Adults—Quick Reference Tool for Professionals. 2003.

[21] Statistics Canada . Low‐income measures thresholds (LIM‐AT and LIM‐BT) for private households of Canada, 2015 https://www12.statcan.gc.ca/census-recensement/2016/ref/dict/tab/t4_2-eng.cfm. Published 2016.

[22] National High Blood Pressure Education Program Working Group on High Blood Pressure in Children and Adolescents. The fourth report on the diagnosis, evaluation, and treatment of high blood pressure in children and adolescents. Pediatrics. 2004;114:555‐576. 15286277

[23] Flynn JT , Kaelber DC , Baker‐Smith CM , et al. Clinical practice guideline for screening and management of high blood pressure in children and adolescents. Pediatrics. 2017;140: e20171904 10.1542/peds.2017-1904 28827377

[24] Anderson LN , Maguire JL , Lebovic G , et al. Duration of fasting, serum lipids, and metabolic profile in early childhood. J Pediatr. 2017;180:47‐52.e1. 10.1016/j.jpeds.2016.09.005 27742126

[25] Expert Panel on Integrated Guidelines for Cardiovascular Health and Risk Reduction in Children and Adolescents: summary report. Pediatrics. 2011;128:S213‐S256. 10.1542/peds.2009-2107C 22084329 PMC4536582

[26] Tremblay MS , LeBlanc AG , Carson V , et al. Canadian Sedentary Behaviour Guidelines for the Early Years (aged 0–4 years). Appl Physiol Nutr Metab. 2012;37:370‐380. 10.1139/h2012-019 22448609

[27] Department of Health . Australia's Physical Activity and Sedentary Behaviour Guidelines. Physical Activity and Sedentary Behaviour. http://www.health.gov.au/internet/main/publishing.nsf/content/health-pubhlth-strateg-phys-act-guidelines. Published November 21, 2017.

[28] Tremblay MS , Carson V , Chaput J‐P , et al. Canadian 24‐Hour Movement Guidelines for Children and Youth: An Integration of Physical Activity, Sedentary Behaviour, and Sleep. Appl Physiol Nutr Metab Physiol Appl Nutr Metab. 2016;41:S311‐S327. 10.1139/apnm-2016-0151 27306437

[29] Hirshkowitz M , Whiton K , Albert SM , et al. National Sleep Foundation's updated sleep duration recommendations: final report. Sleep Health. 2015;1:233‐243. 10.1016/j.sleh.2015.10.004 29073398

[30] Heyman MB , Abrams SA . Fruit juice in infants, children, and adolescents: current recommendations. Pediatrics. 2017;139:e20170967 10.1542/peds.2017-0967 28562300

[31] Ulbricht CM , Chrysanthopoulou SA , Levin L , Lapane KL . The use of latent class analysis for identifying subtypes of depression: a systematic review. Psychiatry Res. 2018;266:228‐246. 10.1016/j.psychres.2018.03.003 29605104 PMC6345275

[32] Muthén B , Muthén LK . Integrating person‐centered and variable‐centered analyses: growth mixture modeling with latent trajectory classes. Alcohol Clin Exp Res. 2000;24:882‐891. 10888079

[33] Carsley SE , Anderson LN , Plumptre L , Parkin PC , Maguire JL , Birken CS . Severe obesity, obesity, and cardiometabolic risk in children 0 to 6 years of age. Child Obes. 2017;13:415‐424. 10.1089/chi.2017.0004 30418801

[34] Sarker H , Anderson LN , Borkhoff CM , et al. Validation of parent‐reported physical activity and sedentary time by accelerometry in young children. BMC Res Notes. 2015;8:735 10.1186/s13104-015-1648-0 26621253 PMC4666154

[35] Bleich SN , Vercammen KA , Zatz LY , Frelier JM , Ebbeling CB , Peeters A . Interventions to prevent global childhood overweight and obesity: a systematic review. Lancet Diabetes Endocrinol. 2018;6:332‐346. 10.1016/S2213-8587(17)30358-3 29066096

