# Supplementary material for: Latent class analysis of obesity‐related characteristics and associations with body mass index among young children
Source: Obes Sci Pract. 2020 Apr 7;6(4):390–400. doi: 10.1002/osp4.414 (PMC7448165; doi:10.1002/osp4.414)
Supplement: Supplementary file 1 — Table S1: Model fit indices for 1 to 8 class LCA models Table S2: Item response probabilities for the six class model overall (male and female children combined) Table S3: Model fit indices for 1 to 8 class LCA models for males only Table S4: Item response probabilities for the four‐class model for males only Table S5: Model fit indices for 1 to 8 class LCA models for females only Table S6: Item response probabilities for the four‐class model for females only [file OSP4-6-390-s001.pdf]

Latent class analysis of obesity-related characteristics and associations with body mass index: A cross-sectional study among children 3-11 years of age

Laura N. Anderson, Ravinder Sandhu, Charles D.G. Keown-Stoneman, Vanessa De Rubeis, Cornelia M. Borkhoff, Sarah Carsley, Jonathon L. Maguire, Catherine S. Birken

Corresponding author:
Laura N. Anderson, Department of Health Research Methods, Evidence, and Impact, McMaster University, CRL-221, 1280 Main Street West, Hamilton, ON Canada L8S 4K1 phone: 905-525-9140 x 21725; email: LN.Anderson@mcmaster.ca

SUPPORTING INFORMATION

Supplemental Table 1: Model fit indices for 1 to 8 class LCA models

Classes	AIC	BIC	CAIC	ABIC
1	7198.6	7296.9	7311.9	7249.2
2	5481.3	5684.5	5715.5	5586.0
3	4577.7	4885.7	4932.7	4736.4
4	4360.4	4773.3	4836.3	4573.1
5	4158.5	4676.2	4755.2	4425.2
6	4012.8	**4635.4**	**4730.4**	4333.5
7	3921.6	4649.1	4760.1	4296.3
8	3891.0	4723.3	4850.3	4319.7

AIC- Akaike information criterion, BIC-Bayesian information criterion, cAIC- consistent Akaike information criterion and a-BIC adjusted Bayesian information criterion. The latent class model that was selected based on the lowest BMI and CAIC is bolded.

Supplemental Table 2: Item response probabilities for the six class model overall (male and female children combined)

**Type of Indicator**	Latent Class Indicators	Class 1 (20%) "Family and Health Risk Behaviors"	Class 2 (7%) "Metabolic Risk"	Class 3 (6%) "High Risk"	Class 4 (21%) "High Triglycerides"	Class 5 (22%) "Health Risk Behaviors and Developmental Concerns"	Class 6 (24%) "Healthy"
**Family Risk Factors**	Family history of cardiometabolic disorders	0.23	0.25	0.30	0.18	0.19	0.14
	Parental BMI>25	0.63	0.46	0.49	0.39	0.47	0.27
	Neighborhood income < $50,000	0.47	0.30	0.33	0.32	0.40	0.28
**Metabolic Risk**	High blood pressure (systolic or diastolic)	0.21	0.19	0.25	0.26	0.23	0.18
	HDL below recommendations (<1.17 mmol/L)	0.10	0.20	0.32	0.44	0.21	0.12
	LDL above recommendations (>2.85 mmol/L)	0.01	1.00	0.57	0.00	0.01	0.00
	Non-HDL above recommendations (>3.11 mmol/L)	0.00	1.00	1.00	0.38	0.00	0.02
	Triglycerides not meeting recommendations (age-based)	0.41	0.59	0.84	1.00	0.49	0.46
**Lifestyle Risk**	Physical Activity not meeting guidelines (age-based)	0.77	0.82	0.79	0.84	0.85	0.79
	Screen time not meeting guidelines (age-based)	0.67	0.43	0.51	0.49	0.52	0.17
	Time spent sleeping below recommendations (age-based)	0.24	0.10	0.16	0.09	0.11	0.04
	SSB intake not meeting guidelines (age-based)	0.74	0.37	0.44	0.42	0.45	0.19
**Developmental Concern**	Developmental or Learning issue	0.01	0.01	0.24	0.01	0.14	0.01
	Requires extra resource at school	0.05	0.02	0.79	0.05	0.89	0.06
	Concern expressed by school	0.17	0.04	0.99	0.13	0.98	0.14

Supplemental Table 3: Model fit indices for 1 to 8 class LCA models for males only

Classes	AIC	BIC	CAIC	ABIC
1	4658.8	4747.4	4762.4	4699.8
2	3723.3	3906.3	3937.3	3807.9
3	3302.5	3580.1	3627.1	3430.8
4	3170.3	3542.4	3605.4	3342.2
5	3092.8	3559.3	3638.3	3308.3
6	3004.3	3565.4	3660.4	3263.5
7	2963.3	3618.9	3729.9	3266.2
8	2928.8	3678.9	3805.9	3275.3

AIC- Akaike information criterion, BIC-Bayesian information criterion, cAIC- consistent Akaike information criterion and a-BIC adjusted Bayesian information criterion. The latent class model that was selected based on the lowest BMI and CAIC is bolded.

Supplemental Table 4: Item response probabilities for the four-class model for males only

Type of Indicator	Latent Class Indicators	Class 1 (9%) "Metabolic Risk"	Class 2 (29%) "High Risk"	Class 3 (39%) "Healthy"	Class 4 (23%) "Triglycerides only"
**Family Risk Factors**	Family history of cardiometabolic disorders	0.21	0.21	0.19	0.19
	Parental BMI>25	0.42	**0.51**	0.44	0.37
	Neighborhood income < $50,000	0.28	0.42	0.37	0.29
**Metabolic Risk**	High blood pressure (systolic or diastolic)	0.23	0.22	0.18	0.26
	HDL below recommendations (<1.17 mmol/L)	0.21	0.24	0.05	0.46
	LDL above recommendations (>2.85 mmol/L)	**0.99**	0.00	0.00	0.00
	Non-HDL above recommendations (>3.11 mmol/L)	**0.99**	0.09	0.00	0.32
	Triglycerides not meeting recommendations (age-based)	**0.62**	**0.56**	0.37	**1.00**
**Lifestyle Risk**	Physical Activity not meeting guidelines (age-based)	**0.81**	**0.82**	**0.77**	**0.82**
	Screen time not meeting guidelines (age-based)	0.47	**0.55**	0.43	0.46
	Time spent sleeping below recommendations (age-based)	0.12	0.14	0.11	0.06
	SSB intake not meeting guidelines (age-based)	0.45	0.47	0.45	0.40
**Developmental Concern**	Developmental or Learning issue	0.16	0.17	0.01	0.02
	Requires extra resource at school	0.36	**0.88**	0.05	0.05
	Concern expressed by school	0.49	**0.99**	0.17	0.20

Supplemental Table 5: Model fit indices for 1 to 8 class LCA models for females only

classes	AIC	BIC	CAIC	ABIC
1	4057.5	4144.7	4159.7	4097.0
2	3312.6	3492.8	3523.8	3394.3
3	2851.4	3124.6	3171.6	2975.3
4	2728.9	3095.1	3158.1	2894.9
5	2676.0	3135.1	3214.1	2884.1
6	2632.4	3184.6	3279.6	2882.7
7	2597.9	3243.1	3354.1	2890.4
8	2592.7	3330.9	3457.9	2927.4

AIC- Akaike information criterion, BIC-Bayesian information criterion, cAIC- consistent Akaike information criterion and a-BIC adjusted Bayesian information criterion. The latent class model that was selected based on the lowest BMI and CAIC is bolded.

Supplemental Table 6: Item response probabilities for the four-class model for females only

**Type of Indicator**	Latent Class Indicators	Class 1 (22%) "Metabolic Risk"	Class 2 (22%) "High Risk"	Class 3 (35%) "Healthy"	Class 4 (20%) "Developmental concerns"
**Family Risk Factors**	Family history of cardiometabolic disorders	0.27	0.20	0.14	0.20
	Parental BMI>25	0.43	**0.63**	0.28	0.45
	Neighborhood income < $50,000	0.33	**0.52**	0.26	0.35
**Metabolic Risk**	High blood pressure (systolic or diastolic)	0.19	0.24	0.21	0.28
	HDL below recommendations (<1.17 mmol/L)	0.30	0.22	0.21	0.20
	LDL above recommendations (>2.85 mmol/L)	**0.53**	0.00	0.00	0.05
	Non-HDL above recommendations (>3.11 mmol/L)	**1.00**	0.00	0.00	0.07
	Triglycerides not meeting recommendations (age-based)	**0.81**	**0.52**	**0.60**	**0.51**
**Lifestyle Risk**	Physical Activity not meeting guidelines (age-based)	**0.82**	**0.84**	**0.80**	**0.88**
	Screen time not meeting guidelines (age-based)	0.44	**0.70**	0.21	0.48
	Time spent sleeping below recommendations (age-based)	0.10	0.28	0.06	0.09
	SSB intake not meeting guidelines (age-based)	0.37	**0.73**	0.26	0.42
**Developmental Concern**	Developmental or Learning issue	0.02	0.01	0.00	0.11
	Requires extra resource at school	0.12	0.07	0.04	**0.92**
	Concern expressed by school	0.21	0.13	0.07	**1.00**
